# Inspiratory muscle training in natural bodybuilders: adaptations in diaphragm muscle thickness and maximal strength

**DOI:** 10.3389/fphys.2025.1628146

**Published:** 2025-08-04

**Authors:** Iskender Güler, Coşkun Yılmaz, Hakan Hüseyin Soylu, Mürşit Ceyhun Birinci, Ayla Arslan, Hakan Ocak, Hüseyin Çayir, Korhan Kavuran, Ajlan Saç, Mine Akkuş Uçar, Baykal Karataş, Levent Ceylan

**Affiliations:** ^1^ Faculty of Education, Amasya University, Amasya, Türkiye; ^2^ Kelkit Aydın Doğan Vocational School, Gümüşhane University, Gümüşhane, Türkiye; ^3^ Gumushane Provincial Health Directorate Kelkit District State Hospital Radiology Department, Gümüşhane, Türkiye; ^4^ Faculty of Sport Sciences, Çanakkale Onsekiz Mart University, Çanakkale, Türkiye; ^5^ Agri Ibrahim Cecen University Faculty of Medicine Department of Anatomy, Ağrı, Türkiye; ^6^ Ministry of Education, Samsun, Türkiye; ^7^ Faculty of Sport Sciences, Bitlis Eren University, Bitlis, Türkiye; ^8^ Kirkpinar Faculty of Sports Science, Trakya University, Edirne, Türkiye; ^9^ Faculty of Sport Sciences, Mardin Artuklu University, Mardin, Türkiye; ^10^ Faculty of Sport Sciences, Agri Ibrahim Cecen University, Ankara, Türkiye; ^11^ Faculty of Sport Sciences, Hitit University, Çorum, Türkiye

**Keywords:** bodybuilding, diaphragm muscle thickness, 1RM, resistance training, inspiratory muscle training, physical performance

## Abstract

**Background:**

The effect of inspiratory muscle training on diaphragm muscle thickness (DT) and one repetition maximal (1RM) in professional natural bodybuilders is still unclear. The aim of the study was to investigate the effect of inspiratory muscle training on diaphragm muscle thickness and 1RM in professional natural bodybuilders.

**Methods:**

The study comprised a total of 22 athletes who participated in bodybuilding competitions. Each athlete had undergone a minimum of 5 years of training, with a minimum weekly commitment of 5 hours. Participants were randomly divided into two groups: inspiratory muscle training (IMT) and control (CON). The CON continued their normal training regime, while the IMT group also performed inspiratory muscle training with a 10% weekly increase by setting the resistance setting of the PowerBreathe® Classic device to 40% of the participant’s maximum inspiratory pressure (MIP). Prior to and during the 4-week training period, 1RM bench press measurements and diaphragm muscle thickness measurements were obtained.

**Results:**

In the comparison of 1RM power values before and after training, it was determined that the IMT group (%: 11.20) had 6.3% more post-activation performance enhancement compared to the CON group (%: 4.9) (p < 0.001). In the study, it was determined that a higher level of significant post-activation performance enhancement was obtained in the IMT group compared to the CON group in the diaphragm muscle thickness inspiratory phase (DT ins) and ekspiratory phase (DT eks) parameters at 20.36% and 19.46%, respectively.

**Conclusion:**

In conclusion, we determined that the addition of progressive loading inspiratory muscle training to preparation programmes in natural bodybuilders will improve diaphragm muscle thickness, 1RM physical performance. In particular, it shows that the diaphragm muscle should be considered not only as a muscle that supports respiration, but also as a muscle that contributes to power generation by optimising intra-abdominal pressure.

## 1 Introduction

One of the main physiological factors affecting physical performance efficiency in athletes is the capacity of the respiratory system and the level of effective use of this system (Dempsey et al., 2006). Especially in trained individuals, the determinant effects of respiratory system functions on maximal force production and general athletic physical performance are attracting more and more attention (Harms et al., 2000; Koç et al., 2025). The type and intensity of exercise that shape athletic performance cause important adaptations not only in the skeletal musculature but also in the respiratory muscles (Granata et al., 2018; Bingöl et al., 2025). In this context, strength training increases muscle fibre contractile capacity and force production, while endurance-based training improves fatigue resistance, especially in the diaphragm and intercostal muscles (Scott et al., 2001; Polla et al., 2004).

The diaphragm is the main inspiratory muscle providing approximately 75% of ventilation and is of vital importance for both respiratory and postural stabilisation (Polla et al., 2004; Erail et al., 2022). The muscle fibre distribution in the adult human diaphragm consists of approximately 55% slow twitch (Type I), 21% fast twitch oxidative (Type IIa) and 24% fast twitch glycolytic (Type IIx) fibres. This distribution enables the diaphragm to successfully meet both continuous rhythmic respiration and situations requiring sudden and intense force (Mizuno, 1991; Orrey, 2014). This structural feature of the diaphragm makes it resistant to mechanical and metabolic loading during high-intensity resistance training.

Resistance training, especially with increasing axial loading, requires the diaphragm to function not only as a ventilatory but also as a trunk stabiliser. In this process, respiratory muscle strength increase is directly related to inspiratory capacity and intra-abdominal pressure (Hackett et al., 2013; Çakaloğlu et al., 2025; Koźlenia and Krzysztofik, 2025). The Valsalva manoeuvre, which is performed to maintain trunk stability, increases the active contraction of the diaphragm and creates a significant mechanical stress on this muscle. In the literature, significant relationships have been reported between respiratory muscle strength measures such as maximal inspiratory pressure (MIP) and maximal expiratory pressure (MEP) and maximal lifting performance. This translates into greater endurance and maximal strength output (Hackett et al., 2013; Çelikel et al., 2025).

Due to its high capillarization and short diffusion distance, the diaphragm muscle facilitates more efficient oxygen delivery to metabolically active tissues involved in the metaboreflex, thereby enhancing resistance to fatigue (Polla et al., 2004; Hellyer et al., 2017). These characteristics enable the diaphragm to maintain its functional continuity, particularly during periods of intense physical activity. The structural and functional adaptation potential of the respiratory muscles can be further enhanced through inspiratory muscle training (IMT). Indeed, IMT has been shown to improve physical performance by reducing respiratory muscle fatigue, attenuating the respiratory metaboreflex, and limiting lactate accumulation (Archiza et al., 2018; Fernández-Lázaro et al., 2021; Fernández-Lázaro et al., 2023; Kowalski et al., 2023; Kowalski et al., 2024; Kowalski et al., 2025). These adaptations contribute to greater maximal power output (Çelikel et al., 2025).

In view of the aforementioned information, it is evident that the diaphragm muscle should be regarded as a significant determinant of sporting and physical performance and an ergogenic target, rather than a passive structure that serves solely for respiration. Despite the fact that research has been conducted on the thickness of the diaphragm muscle in healthy individuals (Enright et al., 2006; Cardenas et al., 2018), individuals suffering from various diseases (Jung et al., 2014; Hellyer et al., 2015; Kim et al., 2017), and athletes (Orrey, 2014; Holtzhausen et al., 2018; Erail et al., 2022), the number of studies focusing on this particular muscle, which is one of the most significant muscles in the respiratory system, remains limited. While the positive effects of different training models on the physical performance of professional natural bodybuilders are well documented (Çelikel et al., 2025), No studies have been found on how respiratory muscle training affects diaphragm muscle thickness and 1RM strength. For this reason, the hypothesis of this study is that respiratory muscle training will increase diaphragm thickness and 1RM strength in professional natural bodybuilders. In consideration of this hypothesis, the present study sought to examine the effects of 4-week inspiratory muscle training on diaphragm muscle thickness and 1RM performance in natural bodybuilders.

## 2 Materials and methods

### 2.1 Participants

The study included 22 male athletes with an average of 3.95 ± 1.05 years of experience in natural bodybuilding, who trained more than 5 h a week and participated in national and international professional natural bodybuilding competitions. In the study, data were collected in the period of May-June 2025. The study was designed as a randomised controlled experimental study. Participants were randomly assigned to two separate groups: IMT group and CON group. None of the participants had IMT experience before the experiment. G* Power (version 3.1.9.2; Dusseldorf, Germany) programme was used to determine the required number of participants. The results of the power analysis sampling study showed that the study could be completed with 10 subjects in each group (effect size: 0.80; actual power: 0.89 for 1RM as the outcome measure) (Çelikel et al., 2025). To eliminate potential problems, 11 participants were included in each group. The numbers from 1 to 22 were placed in sealed envelopes and the participants were asked to select them, and then randomly assigned to two groups by a computerized program to determine which group the subjects forming the sample would be included in (https://www.randomizer.org/).

In the study, all participants underwent the same training program (Table 1) to exclude the contralateral effect and to determine the difference in training load between CON and IMT groups resulting from the IMT intervention (Manca et al., 2017; Kowalski et al., 2025). Individuals who did not meet the following criteria were excluded: (a) being a professional natural bodybuilder with less than 3 years of experience, (b) having any chronic and acute respiratory disease, (c) taking any prescription medication that could affect their response to exercise (d) individuals who were current smokers or had a history of smoking within the past year; and (e) participants who had participated in similar studies within the past 6 months were excluded. All participants signed a waiver declaring their compliance with the World Anti-Doping Agency Code. Before starting the study, all participants were asked to give verbal and written informed consent.

**TABLE 1:** Weekly resistance training program.

Day	Focus area	Warm-up	Summary of exercises	Cardio/Notes
Monday	Lower Body (Strength + Power)	20 min elliptical + 5–10 min dynamic	Squat, Deadlift, Lunge, Calf Raise, Hamstring Curl	20 min elliptical warm-up
Tuesday	Upper Body (Push-Pull)	5–10 min dynamic	Bench Press, Cable Row, Row, Overhead Press, Pull-up, Lateral Raise	20 min low-intensity cycling (cool-down)
Wednesday	Cardiovascular Endurance	5–10 min dynamic	—	30 min interval running or cycling
Thursday	Lower Body (Functional & Core)	20 min elliptical + 5–10 min dynamic	Step-up, Glute Bridge, Lunge, Core Circuit (Plank, Russian Twist, Leg Raise)	20 min elliptical warm-up
Friday	Upper Body (Hypertrophy + Core)	5–10 min dynamic	Incline DB Press, Cable Row, Bench Press, Triceps Pushdown, Biceps Curl, Core Superset	20 min low-intensity cycling (cool-down)
Saturday	Active Recovery/Cardio	5–10 min dynamic	—	30 min brisk walking, cycling
Sunday	Passive Rest			

### 2.2 Experimental design

**FIGURE 1:**
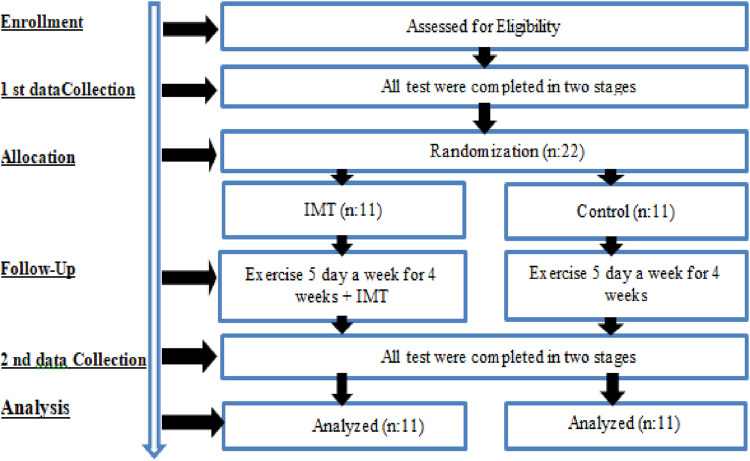
Experimental design.

The natural bodybuilders participating in the study were asked to visit the laboratory environment three times. During the first visit the experimental procedures were introduced and tested. Each subject was provided with a detailed explanation of the IMT procedure. At the second visit 1 week later, 48 h before the training, measurements (1RM, diaphragm thickness by radiologist-guided ultrasonography and pulmonary function tests) were taken and values were recorded. After 48 h of the 4-week training program, final measurements were taken at the third and final visit. All measurements were conducted in a fasted state between 10:00 a.m. and 2:00 p.m. Inspiratory muscle training (IMT) was conducted twice daily (08:00–10:00 a.m. and 03:00–05:00 p.m.). The resistance training (RT) program was performed between 17:00-19:00 on training days (Figure 1). Participants were instructed to abstain from alcohol consumption and high-intensity physical activities for 48 h prior to the baseline measurements as well as prior to the post-intervention assessments following the completion of the 4-week training program. The present study was not previously registered with any clinical trial registry.

### 2.3 Body composition measurement

A professional body composition analysis was conducted using the GAIA 359 Plus, Jawon Medical (Seoul, Korea) device to assess the body composition of the athletes who presented themselves at the laboratory of Gümüşhane University Kelkit Aydın Doğan Vocational School. This device uses a measurement method that generates and calculates information about the tissue according to the type of resistance encountered by low electrical currents as they move between body tissues. Gaia 359 Plus BodyPass was used to determine the height, body weight and body mass index (BMI) of the subjects. Subjects were instructed to stand on the analyser with the entire soles of their bare feet in contact and to remove all outer clothing, including t-shirts and shorts. Subjects were instructed to remove all metal objects before the start of the measurement.

### 2.4 Rate of perceived exertion (RPE)

RPE is a subjective method of measuring an individual’s perception of the physical demands of a particular activity. The most widely used RPE instrument is the Borg scale, a psychophysical, categorical scale with a rating ranging from 6 (no exertion) to 20 (maximum exertion) (Borg et al., 1987).

### 2.5 1 RM estimation method (bench press)

To mitigate the limitations associated with direct 1-repetition maximum (1RM) testing, predictive equations have become increasingly favored in recent years. These models typically rely on submaximal loads within the 2–10 repetition range, which are known to yield more accurate estimations of maximal strength (Mayhew et al., 1995; García-Ramos, 2023). In the present study, 1RM was estimated using Mayhew’s equation, selected for its lower absolute error rate compared to alternative formulas (Pérez-Castilla et al., 2020; Çelikel et al., 2025). The initial load was determined based on each participant’s prior training experience and was selected such that they could complete at least two repetitions or believed they could do so. All participants confirmed familiarity and prior experience with 1RM testing procedures.

The bench press (BP) exercise was chosen for strength assessment due to its relevance in evaluating the functional integration of the respiratory system and upper extremity musculature (Sobierajska-Rek et al., 2022). During the test, participants were positioned on a flat bench with a 90° elbow angle and a 45° shoulder-to-trunk angle. Grip width was standardized according to International Powerlifting Federation (IPF) guidelines. All repetitions were performed with a controlled tempo (V/0/V/0), encompassing deliberate eccentric and concentric phases (Wilk et al., 2020; Qin et al., 2025). The use of wrist straps was prohibited during testing. Participants received standardized verbal encouragement throughout the trials to ensure maximal effort (Weakley et al., 2023). Load (kg) and repetition data were used to calculate estimated 1RM values via Mayhew’s formula.

$${\text{Mayhew}}^{\prime }s\text{ Formula}:\;\left(1\text{RM}=\left(\text{load}/52.5+41.9^{.e-0.055}\;.\text{repetitions}\right)/100\right)$$

### 2.6 Diaphragm thickness (DT) measurement

Diaphragm muscle thickness measurements were performed by a radiologist experienced in musculoskeletal ultrasonography, unaware of group assignments, using a Philips Affiniti 70G ultrasonography device (Philips Healthcare, Bothell, WA, USA) and a 5 cm wide linear transducer probe at a frequency of 12 MHz (Erail et al., 2022). All measurements were performed on the right hemidiaphragm with the participants supine and relaxed. The transducer was placed on the mid-axillary line on the right side in the coronal plane, allowing visualisation through the liver window. Diaphragm thickness was measured at the apposition site of the right haemidiaphragm. Ultrasonographic measurements were conducted in two phases. In the first phase, participants were instructed to exhale as deeply as possible to achieve maximal expiration, followed by breath-holding. During this phase, the diaphragm muscle was visualized, and its thickness was measured. To obtain measurements during the maximal expiratory phase (DTexp), the intercostal space between the eighth and ninth ribs was identified, allowing for optimal diaphragm imaging through the liver window (Hellyer et al., 2017). In the second phase, participants were asked to inhale maximally and hold their breath again, enabling measurement during inspiration. For the maximal inspiratory phase (DTins), the intercostal space between the 10th and 11th ribs was used to determine the optimal imaging site. During the diaphragm thickness measurements, only the data of the muscle tissue were taken into account; echogenic lines formed by the pleura and peritoneum were not included in the measurement. Measurements were repeated three times for each phase and the average of the obtained values was used in the analyses (Erai, 2022). The intraclass correlation coefficient (ICC) was calculated as DKins (0.856) and DKeks (0.877).

### 2.7 Inspiratory muscle training (IMT)

Inspiratory muscle training (IMT) was conducted using the POWERbreathe® device (IMT Technologies Ltd., UK) twice daily (08:00–10:00 a.m. and 03:00–05:00 p.m.), 5 days per week for 4 weeks. Each session included 30 breaths at 40% of the participant’s maximal inspiratory pressure (MIP), which was increased weekly by 10% (Erail et al., 2022; Çelikel et al., 2025). Evidence suggests that a 4-week IMT period applied in the study resulted in an improvement in diaphragm muscle thickness (Erail et al., 2022). MIP was measured using a portable pressure meter (MicroRPM, CareFusion, UK) in accordance with ATS/ERS guidelines (2002), with three seated trials performed from residual volume and the mean value recorded (Yılmaz et al., 2025). The intraclass correlation coefficient (ICC) was calculated as MIP (0.938). All sessions were supervised by a certified coach. The experimental group followed the IMT protocol in addition to their standard training, while the control group maintained only the standard porgramme (Table 1).

### 2.8 Weekly training program

The resistance training (RT) program was designed in alignment with the National Strength and Conditioning Association (NSCA) guidelines to optimize athletic performance. Both groups followed identical training regimens developed and supervised by an experienced senior coach. Training sessions were conducted 5 days per week, with each exercise performed in sets lasting up to 45 s and separated by 30-s rest intervals (Çelikel et al., 2025). Prior to each session, participants completed a 5–10-min dynamic warm-up. To maintain engagement and reduce monotony, exercise routines were varied weekly. The training began with loads corresponding to 50% of each participant’s maximal strength, performed in three sets per exercise. The load was progressively increased by 15% in the second and third sets. All training loads were systematically recorded throughout the study. The program also included two weekly 30-min cardiovascular sessions. On lower-body training days, participants completed a 20-min warm-up on an elliptical trainer before resistance exercises (Table 1). On non-leg training days, a 20-min low-intensity cycling session was incorporated post-workout as a recovery modality (Hackett, 2022; Curovic, 2025). During each training session, the participants were verbally motivated and engaged by senior coach.

### 2.9 Nutrition protocol

Participants’ daily energy requirements were estimated using the Cunningham equation to calculate basal metabolic rate (BMR) (Cunningham, 1991). To account for the thermic effect of food, the BMR was multiplied by a factor of 1.15. This value was then adjusted using a physical activity level coefficient of 1.25, reflecting the participants’ moderately active lifestyle. To ensure a positive energy balance, the resulting total was further increased by 10% (multiplied by 1.10), yielding the final individualized daily energy requirement. Based on these calculations, a personalized 4-week nutritional program was developed for each participant by a certified dietitian. Weekly body weight measurements were used to monitor progress and make adjustments to caloric intake when necessary. The dietitian provided ongoing support, including weekly coaching, food selection guidance, and recipe recommendations. Dietary intake was tracked using the MyFitnessPal application (San Francisco, CA), with participants instructed to log all meals and snacks immediately after consumption, including time stamps. The average daily energy intake ranged from 3,750 to 4,500 kcal (Kim et al., 2011). Macronutrient distribution was standardized across participants, consisting of approximately 64.2% carbohydrates, 27.1% protein, and 8.7% fat. Protein intake was specifically set at 2.2 g per kg of body weight per day. Meals were distributed evenly throughout the day at average intervals of 3.5 h.

### 2.10 Statistical analysis

Statistical analyses were performed via SPSS (Version 21.0 for Windows, Chicago, IL, USA) software, with the statistical significance set at 0.05. The Shapiro‒Wilk normality test was performed to determine the homogeneity of the sample. Repeated measures two-way analysis of variance and Bonferroni correction were used to analyze differences in 1RM and diaphragm thickness measurements between trials. Furthermore, the effect size in pairwise group comparisons was calculated using partial eta-squared (η _p_^2^). The interpretation of the parameter η _p_^2^ is as follows: small values, such as 0.01, indicate a small effect size; medium values, such as 0.06, indicate a medium-sized effect; and large values, such as 0.14, indicate a strong effect (Richardson, 2011). The intraclass correlation coefficient (ICC) was calculated for the purpose of assessing the reproducibility of measurements obtained from the same observer. The interpretation of the results is as follows: scores between 0.50 and 0.75 are considered moderate, scores between 0.75 and 0.90 are considered good, and scores of 0.90 and above are considered excellent (Koo and Li, 2016).

## 3 Results

When examining the group averages of the study participants, it was determined that the IMT group had a mean age of 22.45 ± 4.06 years, a body weight of 80.68 ± 13.47 kg, a height of 178.64 ± 5.84 cm, and 3.91 ± 1.04 years of training experience. In comparison, the control group had a mean age of 24.82 ± 3.66 years, a body weight of 80.73 ± 7.46 kg, a height of 176.91 ± 4.78 cm, and 4.00 ± 1.10 years of training experience (Table 2).

**TABLE 2:** Descriptive.

Descriptive	IMT (n:11)		Control (n:11)	
	X±S.D		X±S.D	
Age (Year)	22,45 ± 4,06		24,82 ± 3,66	
Weight (kg)	80,68 ± 13,47		80,73 ± 7,46	
Height (cm)	178,64 ± 5,84		176,91 ± 4,78	
Experience (Year)	3,91	1,04	4,00	1,10

**FIGURE 2:**
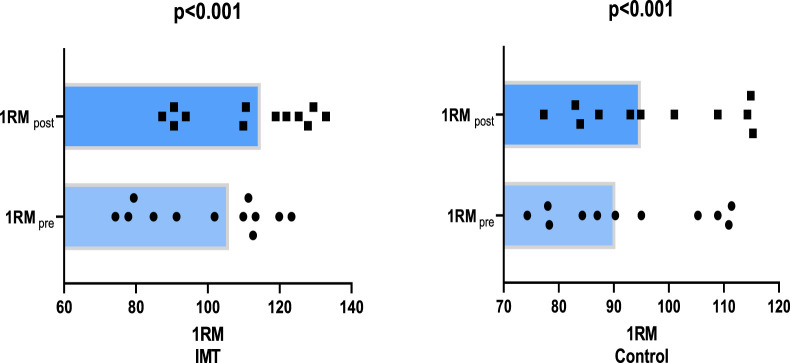
Comparison of mean values of 1RM before and after training.

A subsequent comparison of 1RM power values before and after training revealed a 6.3% greater improvement in the IMT group (11.20%, p < 0.001, Figure 2) compared to the control group (4.9%, p < 0.001, Figure 2). The addition of IMT to the 4-week training programme was found to have a significant effect on 1RM power output (p < 0.001, η _p_^2^ = 0.716).

**FIGURE 3:**
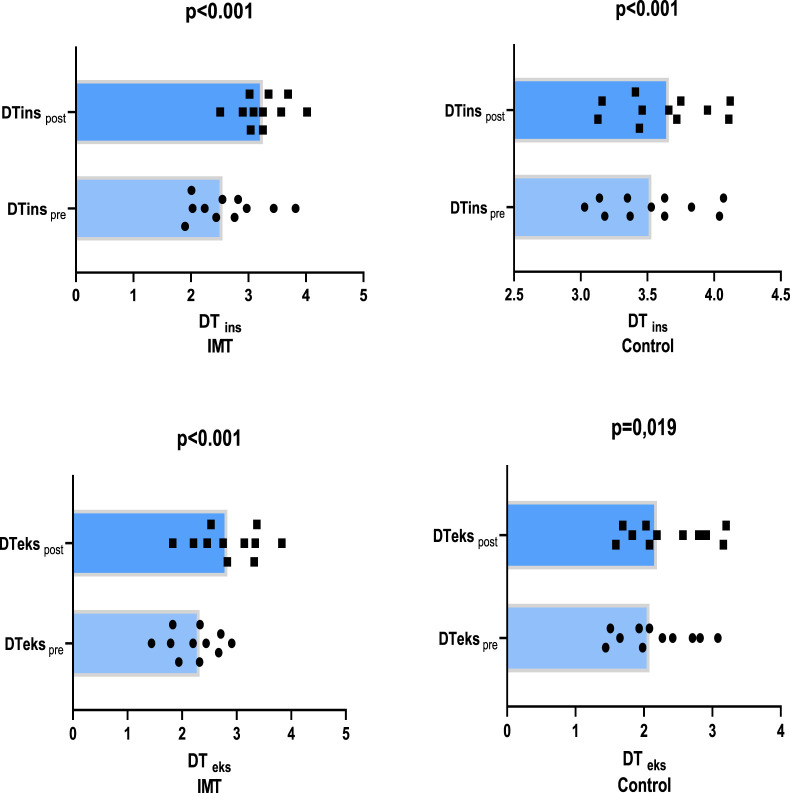
Comparison of mean values of diaphragm muscle thickness before and after training.

In the study, comparison of Diaphragm inspiratory phase (DT_ins_) values, it was determined that the IMT group (23.19%, p < 0.001) had a 20.36% more improvement than the control group (2.83%, p < 0.001) (Figure 3). The addition of IMT to the 4-week training programme was found to have a small effect on DKins thickness (p < 0.001, η _p_^2^. = 0.435). In the comparison of Diaphragm expiratory phase (DT_eks_) values, it was determined that there was a 19.46% more improvement in the IMT group (28.69%, p < 0.001) compared to the control group (9.21%, p = 0.019) (Figure 3). The addition of IMT to the 4-week training programme was found to have a small effect on DKeks thickness (p = 0.003, η _p_^2^ = 0.661).

**FIGURE 4:**
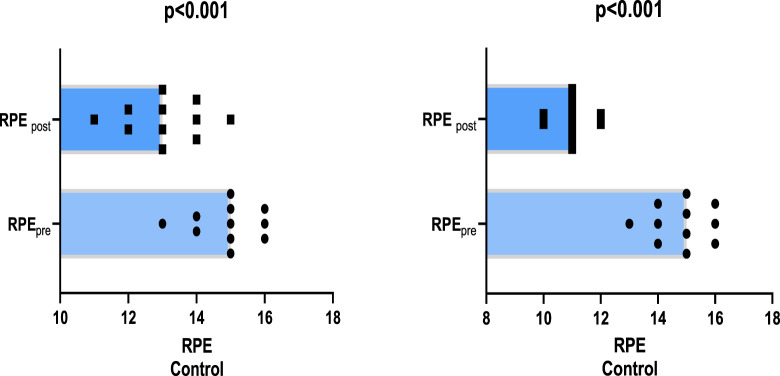
Comparison of mean values of rate of perceived exertion (RPE) before and after training.

In the comparison of RPE values before and after the 4-week training period, it was determined that there was a 13.57% greater decrease in the IMT group (25.77%, p < 0.001) compared to the control group (12.20%, p < 0.001) (Figure 4)). The addition of IMT to the 4-week training programme was found to have a medium effect on RPE thickness (p = 0.008, η _p_^2^ = 0.780).

## 4 Discussion

The primary aim of this study was to examine the effects of respiratory muscle training on diaphragm muscle thickness and one repetition maximum (1RM) performance in natural bodybuilders. There are studies investigating the effects of respiratory muscle training on 1RM performance in professional natural bodybuilders (Çelikel et al., 2025). However, to the best of our knowledge, this is the first study to consider both diaphragm muscle thickness and 1RM performance. The main findings of the study showed that in the IMT group, after a 4-week training period, significant improvements in 1RM, DTins and DTeks, RPE parameters were obtained at higher levels of 6.3% (p < 0.001, η _p_^2^ = 0.716), 20.36% (p < 0.001, η _p_^2^. = 0.435), 19.46% (p = 0.003, η _p_^2^ = 0.661) and 13.57% (p = 0.008, η _p_^2^ = 0.780), respectively, compared to the control group.

Studies show that different training protocols such as respiratory muscle training, isometric muscle movements and integrated neuromuscular training have significant effects on 1RM performance. Çelikel et al. (2025) reported that respiratory muscle training provided positive effects on 1RM in professional bodybuilders. Similarly, Koźlenia and Krzysztofik (2025), in their isometric muscle movements study on bodybuilders, emphasized that although no change in 1RM value was observed with moderate submaximal load use, upper body power output (bench press) was significantly increased. Liu et al. (2025) found that integrated neuromuscular training (INT) provided a significant improvement in 1RM bench press and squat performance of military personnel compared to traditional physical training. These findings demonstrate the effects of different types of training on strength development and 1RM performance and suggest that programs such as respiratory muscle training, isometric muscle movements, and integrated neuromuscular training provide significant improvements, especially on force production and maximal power output. The implementation of such protocols offers an important strategy to increase the strength capacity of individuals and optimize their performance.

The application of high-intensity loads during bodybuilding training has been demonstrated to induce a substantial physiological stress, particularly on the diaphragm muscle (Brown et al., 2013). This physiological stress has been shown to promote significant adaptations in the respiratory and postural functions of the diaphragm muscle (Niewiadomski et al., 2012; Hackett, 2020; Çelikel et al., 2025). It is important to note that diaphragm thickness and inspiratory muscle strength are related (Erail et al., 2022). The downward movement of the diaphragm, in conjunction with a strong contraction of these muscles, results in an increase in intra-abdominal pressure. This is due to the application of pressure to the intra-abdominal organs (Holtzhausen et al., 2018). This phenomenon is further substantiated by the concomitant activation of the abdominal muscles, which contributes to trunk stabilization (Martuscello et al., 2013; Cavaggioni et al., 2015). In this process, the diaphragm not only maintains respiratory function, but also acts as a key stabilizer in the regulation of intra-abdominal pressure. Consequently, it is exposed to a meaningful training stimulus through repetitive heavy loading. According to the extant literature, substantial adaptations in strength and endurance occur in the diaphragm muscle exposed to elevated transdiaphragmatic pressures (DePalo et al., 2004). However, the directness of this relationship may vary depending on factors such as the specific training protocol employed, individual physiological characteristics, and synergistic interactions among muscle groups. Therefore, further well-controlled studies are warranted to elucidate the impact of diaphragm thickness on maximal strength performance.

Moreover, bodybuilding training has been shown to result in more than just structural changes, such as hypertrophy in the skeletal muscle system. It has also been demonstrated to directly impact the diaphragm muscle through mechanisms such as the Valsalva maneuver (Abe et al., 2000; Çelikel et al., 2025). The Valsalva maneuver, particularly during the execution of loads exceeding 75% of one repetition maximum, necessitates the active contraction of the diaphragm, thereby increasing intra-abdominal pressure. In this process, the diaphragm plays a dual role in regulating respiration and ensuring spinal stability (Welch et al., 2019). The observation that the activation level of the diaphragm varies in exercises that demand relatively lower spinal stability, such as the bench press, suggests that the impact of this muscle on bodybuilding performance may be contingent on the nature of the exercise (MacDougall et al., 1992). Moreover, it has been documented that the impact of the diaphragm muscle on limb exercise performance is influenced by the distribution of peripheral blood flow, which undergoes alterations in accordance with diaphragm movements (Enright et al., 2006; Welch et al., 2018). This circulatory effect of has the potential to become a performance-limiting factor, especially during prolonged or high-intensity exercise (McConnell and Lomax, 2006).

In order to develop a comprehensive understanding of the role of the diaphragm in heavy lifting, it is necessary to analyze its movement in four distinct phases. During the eccentric phase, such as the movement of lowering the weight during a bench press, the breath is inhaled, causing the diaphragm to contract and move downward. During this phase, the intra-abdominal pressure is relatively low (Debernardi et al., 2024). Prior to the concentric phase (phase two), the athlete typically takes a deep breath, holds the breath, and performs the Valsalva maneuver (Brown et al., 2013). This results in a significant increase in intra-abdominal pressure while the diaphragm remains contracted. In the third phase, the concentric phase (weight lifting), the diaphragm remains contracted and the intra-abdominal pressure reaches its maximum. This approach is designed to enhance spinal stability by providing mechanical support to the spine. In the final phase (locking and exhalation), once the movement is complete, the diaphragm relaxes, moves upwards, and the pressure returns to normal as air is expelled (Seo et al., 2024). A comprehensive examination of the available literature reveals that the diaphragm muscle is not merely a passive structure associated with the respiratory system. Rather, it performs an active and adaptive role in high-intensity exercises, such as weightlifting (Sembera et al., 2022; Debernardi et al., 2024, Seo et al., 2024). In this context, it is proposed that the physiological stimuli generated by bodybuilding training may induce substantial adaptations in the diaphragm muscle, thereby underscoring the significance of this muscle as a critical component associated with performance.

A paucity of studies has been conducted on the effects of respiratory muscle training on diaphragm muscle thickness and 1RM in professional natural bodybuilders. This finding can be regarded as a significant strength of the present study. The present study is not without its limitations. The number of participants was constrained by the fact that they were high-performance male natural bodybuilders. The limited sample size precluded the ability to generalize the findings. A larger sample size may provide more accurate data. The evaluation process incorporated two primary assessments: one-repetition maximum (1RM) and ultrasonography. The study’s duration, spanning a mere 4 weeks, precludes the assessment of long-term outcomes, thereby impeding the comprehension of sustained effects. Additionally, the study did not address potential gender differences. In the future, researchers should endeavor to include larger and more diverse samples in their studies. Furthermore, they should examine long-term effects, address gender as a variable, and explore a wider range of exercise protocols.

## 5 Conclusion

The central hypothesis of this study, which suggested that inspiratory muscle training (IMT) would improve 1RM performance by increasing diaphragm muscle thickness in natural bodybuilders, was confirmed. Athletes who incorporated IMT into their training regimens showed greater improvements in 1RM and diaphragm muscle thickness compared to those who adhered to traditional training protocols. In light of these findings, it is recommended that IMT be incorporated into training programs for professional natural bodybuilders.

## Data Availability

The datasets presented in this study can be found in online repositories. The names of the repository/repositories and accession number(s) can be found in the article/supplementary material.
